# Using Nonexperts for Annotating Pharmacokinetic Drug-Drug Interaction Mentions in Product Labeling: A Feasibility Study

**DOI:** 10.2196/resprot.5028

**Published:** 2016-04-11

**Authors:** Harry Hochheiser, Yifan Ning, Andres Hernandez, John R Horn, Rebecca Jacobson, Richard D Boyce

**Affiliations:** ^1^ Department of Biomedical Informatics School of Medicine University of Pittsburgh Pittsburgh, PA United States; ^2^ Intelligent Systems Program University of Pittsburgh Pittsburgh, PA United States; ^3^ Center for Bioinformatics and Computational Biology BIOS Manizales Colombia; ^4^ Department of Pharmacy School of Pharmacy and University of Washington Medicine, Pharmacy Services University of Washington Seattle, WA United States

**Keywords:** crowdsourcing, natural language processing, drug interactions, drug product labeling, structured product labels

## Abstract

**Background:**

Because vital details of potential pharmacokinetic drug-drug interactions are often described in free-text structured product labels, manual curation is a necessary but expensive step in the development of electronic drug-drug interaction information resources. The use of nonexperts to annotate potential drug-drug interaction (PDDI) mentions in drug product label annotation may be a means of lessening the burden of manual curation.

**Objective:**

Our goal was to explore the practicality of using nonexpert participants to annotate drug-drug interaction descriptions from structured product labels. By presenting annotation tasks to both pharmacy experts and relatively naïve participants, we hoped to demonstrate the feasibility of using nonexpert annotators for drug-drug information annotation. We were also interested in exploring whether and to what extent natural language processing (NLP) preannotation helped improve task completion time, accuracy, and subjective satisfaction.

**Methods:**

Two experts and 4 nonexperts were asked to annotate 208 structured product label sections under 4 conditions completed sequentially: (1) no NLP assistance, (2) preannotation of drug mentions, (3) preannotation of drug mentions and PDDIs, and (4) a repeat of the no-annotation condition. Results were evaluated within the 2 groups and relative to an existing gold standard. Participants were asked to provide reports on the time required to complete tasks and their perceptions of task difficulty.

**Results:**

One of the experts and 3 of the nonexperts completed all tasks. Annotation results from the nonexpert group were relatively strong in every scenario and better than the performance of the NLP pipeline. The expert and 2 of the nonexperts were able to complete most tasks in less than 3 hours. Usability perceptions were generally positive (3.67 for expert, mean of 3.33 for nonexperts).

**Conclusions:**

The results suggest that nonexpert annotation might be a feasible option for comprehensive labeling of annotated PDDIs across a broader range of drug product labels. Preannotation of drug mentions may ease the annotation task. However, preannotation of PDDIs, as operationalized in this study, presented the participants with difficulties. Future work should test if these issues can be addressed by the use of better performing NLP and a different approach to presenting the PDDI preannotations to users during the annotation workflow.

##  Introduction

 Exposure to interacting drug combinations can lead to patient harm. Recent estimates indicate that between 5.3% and 14.3% of hospital patients in the United States experience a clinically meaningful alteration in the exposure or response of one drug occurring as a result of coadministration of another drug [1]. Fortunately, such harm can often be avoided by employing appropriate management strategies [2]. Toward that goal, US federal regulations require the mention of known, clinically relevant potential drug-drug interactions (PDDIs) in prescription drug labeling [3,4].

Structured product labels (SPLs) are mandated by the US Food and Drug Administration. The labels, produced by pharmaceutical manufacturers, are presented in a standardized format [5] and approved by regulators. As detailed descriptions subject to regulatory approval, SPLs play a vital role in disseminating drug information. However, the structure in these documents is only in the form of high-level sections such as *Description*, *Indications and Usage*, *Contraindications*, and *Warnings*. Specific PDDI details are given in plain text, tables, and figures within the *Drug Interactions* section or other locations throughout the label. Although future efforts may lead to more structured and therefore more computable labels, the regulatory importance of the SPLs and the legacy labels of more than 16,000 drugs make the labels key resources for drug-drug interaction information.

Unfortunately, product labeling is incomplete. A study of drugs that interact with the narrow therapeutic range drug warfarin found PDDI information deficiencies in 15% of relevant product labels [6]. A broader study of drugs sold in the United States, United Kingdom, and Germany found that a warning about a critical drug interaction was missing from the label of one of the interacting drugs at least 40% of the time [7]. Although publicly available PDDI information sources can serve as useful adjuncts to product label information, these collections are often far from complete. Our recent analysis of 14 collections of PDDI information found significant divergence, with overlap between pairs of sources usually less than 50% [8]. Addressing the issue of missing product label PDDI information is important to better meet the information needs of drug experts, clinicians, and patients.

We hypothesize that a computable representation of PDDIs present in product labels and other high-quality sources will enable novel methods for drug information retrieval that will in turn provide researchers and clinicians with improved capabilities for finding complete and current DDI information. Testing this hypothesis requires an efficient means of generating computable representations of PDDI mentions.

In prior work, we developed a prototype system that used simple named entity recognition (NER) and Semantic Web Linked Data [9] to link claims about PDDIs from publicly available external resources to the *Drug Interactions* section of the product label [10]. Experiments found that our system linked at least one potentially novel interaction (ie, not mentioned in the label) to the *Drug Interactions* section of product labeling for 20 antidepressants. Moreover, there were several cases where all of the PDDI mentions linked to the *Drug Interactions* section for an antidepressant were potentially novel and would complement product label information. For example, an interaction between escitalopram and tapentadol mentioned in the National Drug File-Reference Terminology [11,12] was potentially novel to all 20 escitalopram product labels.

While promising, the simple NER approach often missed potentially important links between the label and other sources. Sophisticated natural language processing (NLP) methods might prove to be more complete, accurate, and scalable than simple NER. However, there is reason to believe that even the best NLP methods would not perform well enough to guarantee automatic identification of all PDDI mentions across all drug product labels. The PDDI NLP algorithm that performed best against the 2013 SemEval Challenge text corpus had a sentence-level recall of 0.81 and a precision of 0.86 (F_1_ =0.84)[13]. An algorithm we developed in prior work focusing specifically on NLP identification of pharmacokinetic PDDI mentions within product label sections had a document level recall of 0.84 and a precision of 0.88 (F_1_=0.86) [14] (sentence-level performance was not evaluated).

Based on these findings, we have concluded that the involvement of human curators is necessary for the task of generating computable representations of PDDIs present in product labels and other high-quality sources. The use of semiautomatic curation is relatively common in biomedicine [15]. Unfortunately, the high cost of expert annotation is a major potential barrier to further progress. New approaches are needed to increase the scale and quality of data curation.

Replacing experts with nonexpert crowds (crowdsourcing) can increase the feasibility of large-scale annotation tasks for biomedical data [16-18]. Initial efforts at crowdsourcing for the annotation of medical text have found the method to be effective when the workflow is properly managed [16]. Results can be comparable in quality to those obtained via more traditional and expensive expert annotation methods [17]. Crowdsourcing is particularly attractive for obtaining results faster and at a lower cost than other participant recruitment schemes [17]. The Informatics for Integrating Biology and the Bedside (i2b2) 2010 workshop assessment found that a well-selected group of nonexperts could perform extraction of drug information from clinical reports [19]. Other biomedical efforts have applied crowdsourcing to gene-mutation mentions in the biomedical literature [20] and for clinical trial announcements [21]. A study of the feasibility of using people recruited through Amazon’s Mechanical Turk to annotate medication indications found that nonexperts could achieve accuracy of greater than 95% on the binary question of whether a medication is an indication for a disease mentioned in the medication’s drug label [22]. Similar approaches have been used to engage communities of experts in tackling challenges such as linking medications and problems in clinical texts from electronic medical records [23,24] and developing mappings between institutional procedure descriptions and Logical Observation Identifiers Names and Codes (LOINC) [25,26].

Our experience with NLP methods for extracting PDDI annotations suggests the possibility of using NLP annotation to provide suggestions to human annotators. Previous efforts have explored the possibility of using such preannotation. Hanauer et al [27] found that iterative alternation between human annotation and model building facilitated rapid creation of NLP models. Some comparative studies have shown that preannotation can improve annotator performance relative to unassisted annotation [28-30], but other studies have seen no difference [31].

The goal of this study was to assess the potential feasibility of using persons who are not drug experts in the task of annotating PDDIs mentioned in drug product labels. A secondary goal was to test the influence of NLP assistance on the annotation quality of both experts and nonexperts.

## Methods

### The Annotation Model

**Figure 1:**
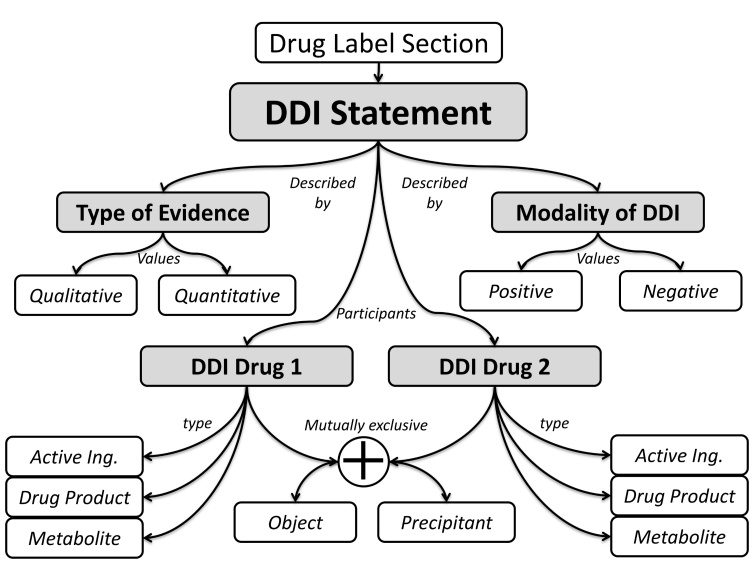
Data model used in this study for PDDIs mentioned within drug product labels.

PDDI annotation requires a data model that describes the types of information that must be collected. The PDDI data model used in this study is given in Figure 1. Each PDDI mention is extracted from a span of sentences present within a product label and can include four features:

- Type of evidence (active ingredient, metabolite, or drug product): an active ingredient is a pharmacologically active chemical component used in a drug product. A metabolite is a biochemical entity produced as a result of drug metabolism. A drug product is a packaging of an active ingredient for sale or distribution, often identified by a brand name. Throughout this paper, we use the generic term “drug” to mean any of these three types.
- Role (object or precipitant): the role that each drug plays within the interaction. In pharmacokinetic PDDIs the precipitant drug affects an enzyme that regulates the absorption, distribution metabolism, or excretion of the object drug.
- Statement (quantitative or qualitative): an indication of whether the PDDI mention describes the pharmacokinetic effect of a DDI in quantitative terms (50% increase) or qualitative terms (increase or decrease) with no indication of magnitude.
- Modality (positive or negative): whether the PDDI mention is making a positive or negative claim. A positive claim is one that supports the existence of the interaction. A negative claim is one that explicitly states that no interaction exists between the drugs in question.

### Natural Language Processing Pipeline and Postprocessing Module

**Figure 2:**
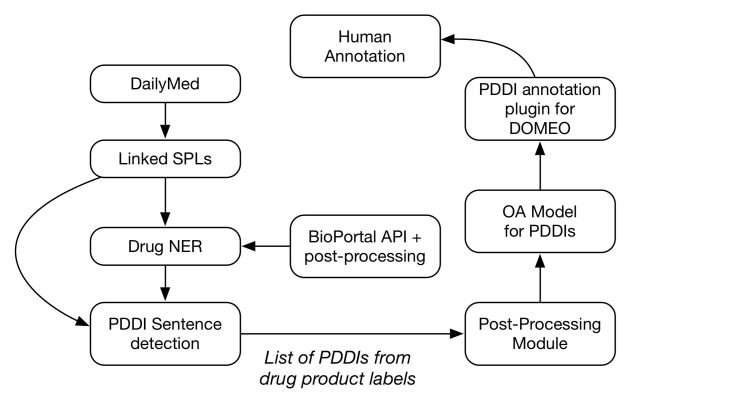
Pipeline for extraction of pharmacokinetic PDDIs from drug labels sections.

In prior work, we developed algorithms for extracting drug named entities and pharmacokinetic PDDI mentions from drug product labels [14]. We integrated our NLP algorithms into a preannotation pipeline (Figure 2). The pipeline used the following steps:

1. The NLP process applies NER to each product label section [32]. The NER algorithm uses the National Center for Biomedical Ontology BioPortal Annotator to extract drug mentions and synonyms from the RxNorm and MeSH terminologies [33]. The results are postprocessed to improve recall and precision by filtering out entities that are not active ingredients, drug products, or metabolites based on entity relationships provided by RxNorm and WordNet [34].
2. Output from the NER process is then processed by an NLP algorithm for identifying pharmacokinetic PDDI mentions [14]. For each product label section, the PDDI extraction algorithm outputs a table of sentence spans labeled as to whether they include a pharmacokinetic PDDI (true or false). Spans including PDDI mentions are also labeled to indicate the modality of the mention (positive or negative). Output of the NLP algorithm is passed to a postprocessing module designed to increase the process's precision and recall (Figure 2). This module uses RxNorm relationships and exact case-insensitive matching to map drug product mentions to unique identifiers of the sole active ingredients.
3. The PDDI mentions present in the corpus are transformed into a machine-readable annotation schema using the Open Annotation data model [35], necessary for subsequent loading into the study annotation tool.
4. Finally, the resulting preannotated named entities and PDDI mentions are loaded into the study annotation tool.

### Reference Standard

In an earlier study, we developed a corpus of 208 annotated PDDI statements from SPLs. Two experts in drug information used the data model described above to annotate these sections, with subsequent discussions used to develop a consensus model. The resulting corpus contains 607 pharmacokinetic PDDI mentions along with 3351 active ingredients, 234 drug products, and 201 metabolite mentions [14]. These sections were used in the current study, with the consensus annotations acting as a gold standard.

### Drug-Drug Interaction Annotation Tool

**Figure 3:**
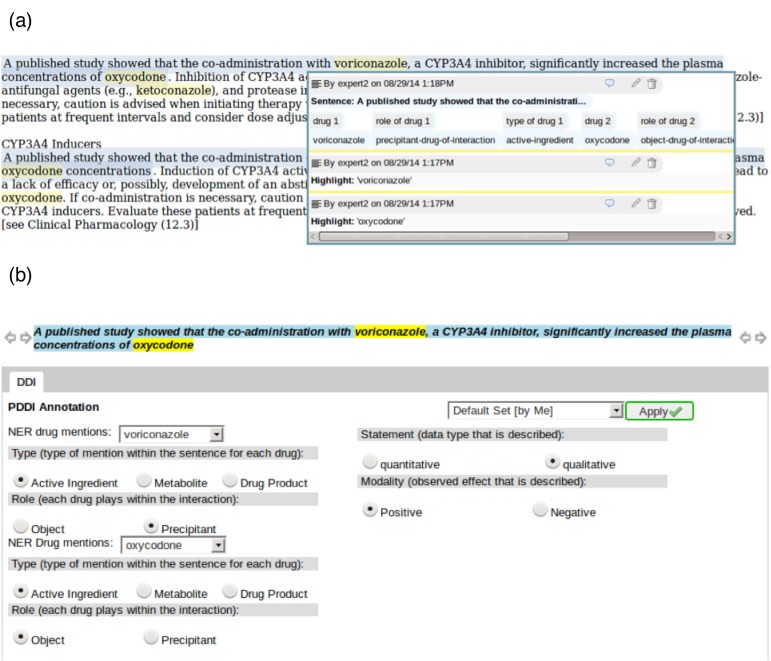
Screenshots of the DOMEO PDDI annotation plugin: (a) product label excerpt with text selected by an annotator as being relevant to a PDDI and (b) form with the fields that the annotator must complete in order to describe the PDDI using the data model described in Figure 1.

Participants used a custom-designed user interface (Figure 3) based on the DOMEO Web-based system [36] to annotate PDDI mentions. DOMEO is an extensible Web application that supports scalable Web-based annotation necessary for crowdsourcing efforts [37]. We extended DOMEO with a plugin that can be used to link text in drug label sections with details of the PDDI data model (Figure 1) [38]. To complete a PDDI annotation task, users would view a product label section and select one or more sentences from the section that discusses the PDDI. They would then use our PDDI annotation plugin to provide values in a Web form indicating the two drugs involved in the interaction, the type and role for each drug, the type of PDDI mention (quantitative or qualitative), and the modality of the mention (positive or negative).

### Annotation Scenarios

We explored four annotation scenarios aimed at assessing the impact of different approaches to NLP preannotation. The 208 product label sections from our reference standard [14] were distributed across four scenarios so that each scenario had roughly the same number of long and short sections:

- Scenario 1 (no assistance) consisted of 52 label sections with no NLP assistance for annotation. Annotators had to read and highlight all drugs and PDDI mentions within the assigned drug label sections.
- Scenario 2 (drug mentions) consisted of 52 drug label sections with preannotations for drug mentions but not PDDI mentions. Annotators had to correct preannotated drug mentions, identify any drug mentions that the NLP missed, and highlight all PDDIs mentioned in the label sentences.
- Scenario 3 (drug mention plus PDDIs) consisted of 53 label sections preannotated with both drug and PDDI mentions. The annotator had to edit and correct NLP preannotations and add any mentions missed by the NLP.
- Scenario 4 (no assistance, second time), a second completely unassisted scenario, was included with the intent of measuring any learning effects associated with the completion of the NLP-assisted tasks. This scenario consisted of 48 drug label sections.

Each participant completed all four scenarios in order. Three of the 208 sections were reserved for training purposes to familiarize participants with the annotation tool and process, leaving 205 sections to be annotated by each participant.

### Participants

A drug expert was defined as a professional in pharmacy or related field with a Doctor of Pharmacy degree or equivalent and more than five years’ experience in drug-drug interaction research. A drug nonexpert was defined as an undergraduate or graduate student with some basic training in chemistry. Both expert and nonexpert participants were recruited from personal contacts of the investigative team. All participants were compensated for participating in this study. The University of Pittsburgh Institutional Review Board approved the study protocol as exempt.

### Annotator Guidelines and Training

Annotators were provided with guidelines describing the annotation task. Guidelines were written based on assumption of college-level formal training in chemistry (eg, general chemistry) for both groups. The complete guidelines are provided in Multimedia Appendix 1. Participants attended a half-day training session that introduced the goal of the annotation task and provided the annotation guidelines.

### Annotation Tasks

Each participant completed all of the four scenarios in the order given above. For each task, the annotators were asked to read the entire content of the relevant drug label sections, identify all drug and PDDI mentions, and record information about the PDDI corresponding to the PDDI annotation model. They were also asked to self-report the amount of time it took to completely annotate each section. The results of each training task were verified to ensure that each annotator completed each scenario according to the study requirements. A short questionnaire completed at the end of each scenario included closed- and open-ended questions about the participant's perception of the usability and effectiveness of the annotation tool and NLP preannotation.

### Annotation Performance Metrics

Performance metrics were calculated by comparing the PDDIs in each participant's results with the reference standard described above [14]. User’s annotations were considered true positives if they (a) matched the precipitant, object, and modality of the reference standard and (b) used sentences that either partially or exactly overlapped the sentences used in the reference standard. Metrics were computed by label and then averaged by scenario.

We supplemented the standard metrics of precision, recall, and F_1_ with additional metrics to gain more insight into the effect of NLP preannotation on the PDDI annotation task. Specifically, for Scenario 3 (ie, the full NER plus NLP preannotation), we evaluated how often participants decided to change NLP annotations and whether those NLP annotations agreed or disagreed with the reference standard.

##  Results

Two experts and 4 nonexperts were recruited into the study. One expert left the study after experiencing too many difficulties with the PDDI annotation user interface. One nonexpert left the study because of not having time to complete annotations due to work and school commitments. The remaining participants completed the annotation task for all scenarios.

**Figure 4:**
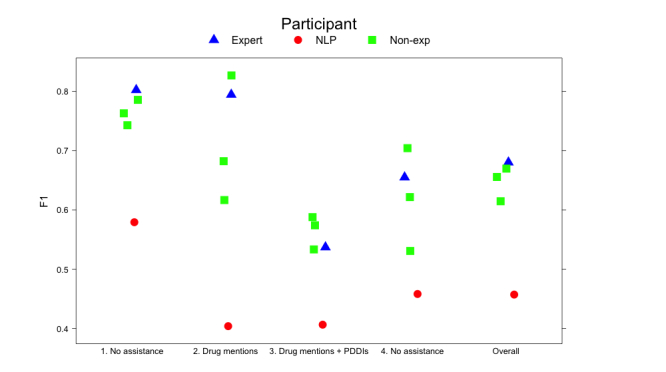
Annotator and NLP performance (F1 scores) for each of the four scenarios and overall performance across all four scenarios.

Annotation performances measured in F_1_ score relative to the reference standard [14] indicate relatively strong performance (F_1_>0.7) for all participants for the first two scenarios, with a drop in performance for the last two scenarios. (Figure 4 and Table 1; full recall and precision results in Multimedia Appendix 2). The performance of the entire NLP pipeline is included for each scenario for comparison even though participants were only provided PDDI preannotations in Scenario 3.

Tables 2 and 3 summarize self-reported task completion times and subjective feedback across the first three scenarios. As Scenario 4 was conducted solely to assess learning effects, task completion time and subjective responses were not collected. Participants differed in their reports of time required, ranging from Nonexpert 1 reporting times comparable to those of the expert to Nonexpert 3 reporting more than 5 hours spent completing Scenario 3 (Table 2).

Results from the subjective question assessing ease of use are given in Table 3. Users agreed that the PDDI annotation interface was moderately difficult when full preannotation assistance was enabled and also agreed that the PDDI annotation plugin without NLP assistance or using a lower level of assistance is relatively easy to use. Full questionnaires and results are given in multimedia appendices 3-7.

Tables 4 and 5 illustrate the agreement between the participants, NLP, and reference standard in the scenario with NLP and preannotation assistance (Scenario 3). Table 4 addresses performance on the 151 PDDI annotations found in the reference standard, while Table 5 summarizes false positives—mentions extracted in the NLP or by users that were not found in the reference standard.

Though exploratory because of the very small sample size, success in detecting true-positive PDDI mentions missed by the NLP was similar between the expert and nonexperts (Table 4, column 2). The expert also had slightly more false negatives than the nonexpert participants irrespective of whether spans were found by NLP (Table 4, columns 1 and 3). False-positive rates for the expert were comparable to those of the nonexperts (Table 5, column 1). Nonexperts also seemed to be slightly more likely to agree with false-positive mentions extracted by the NLP (Table 5, columns 2 and 3).

**Table 1:** F_1_ measures for all participants and NLP system across all scenarios and overall.

Annotator	Scenario 1^a^	Scenario 2^b^	Scenario 3^c^	Scenario 4^d^	Overall
Expert	0.80	0.79	0.54	0.66	0.68
Nonexpert 1	0.79	0.83	0.59	0.53	0.66
Nonexpert 2	0.76	0.68	0.57	0.70	0.67
Nonexpert 3	0.74	0.62	0.53	0.62	0.61
NLP	0.58	0.40	0.41	0.46	0.46

^a^No assistance.

^b^Preannotation of drug mentions.

^c^Preannotation of drug mentions and PDDIs.

^d^No assistance.

**Table 2:** Participant self-reported task completion times.

Participant	Scenario	<1 hour	1-3 hours	3-5 hours	>5 hours
Expert					
	1	X			
	2	X			
	3	X			
Nonexpert 1					
	1	X			
	2	X			
	3	X			
Nonexpert 2					
	1		X		
	2	X			
	3	X			
Nonexpert 3					
	1			X	
	2			X	
	3				X

**Table 3:** Usability questionnaire results. All results reported on a 5-point scale (1=*very difficult* to 5=*very easy*).

Participant	Scenario 1	Scenario 2	Scenario 3	Mean
Expert	2	4	2	2.67
Nonexpert 1	4	5	2	3.67
Nonexpert 2	4	5	2	3.67
Nonexpert 3	3	3	2	2.67
Mean	3.25	4.25	2	—

**Table 4:** Comparison of agreement between the participants, NLP preannotation, and PDDI annotations (N=151) in the reference standard during the scenario with NER and NLP preannotation assistance (Scenario 3).

NLP Result	No mention found		Mention	
Participant	No mention	Mention^a^	No mention^b^	Mention
	NLP FN^f^ User FN n (%)	NLP FN User TP n (%)	NLP TP^c^ User FN n (%)	NLP TP User TP n (%)
Expert	59 (39.1)	50 (33.1)	23 (15.2)	19 (12.6)
Nonexpert 1	46 (30.5)	63 (41.7)	11 (7.3)	31 (20.5)
Nonexpert 2	43 (28.5)	66 (43.7)	11 (7.3)	31 (20.5)
Nonexpert 3	49 (32.5)	60 (39.7)	13 (8.6)	29 (19.2)

^a^Indicates case where the user corrected an NLP error.

^b^Indicates cases where the NLP was correct and the user was incorrect.

^c^FN: false negative

^d^TP: true positive

**Table 5:** Analysis of user and NLP false positives relative to the reference standard for Scenario 3.

NLP result	No mention	Mention (n=93)	
Participant	Mention^a^	No mention^b^	Mention
	NLP TN User FP n	NLP FP User TN n (%)	NLP FP User FP n (%)
Expert	25	93 (100)	0 (0)
Nonexpert 1	16	88 (94.6)	5 (5.4)
Nonexpert 2	37	86 (92.5)	7 (7.5)
Nonexpert 3	24	88 (94.6)	5 (5.4)

^a^Indicates cases where the user identified spans that were not identified by the NLP. ^b^Indicates cases where the NLP identified spans that the participant did not annotate.

## Discussion

### Overview

Our long-term goal is to develop tools that will deliver computable representations of reliable, accurate PDDI information to clinicians, facilitating decision support and hopefully reducing adverse events. The number of drugs that might need to be addressed (more than 16,000) and the complexity of the content in the SPLs make this a daunting task. Our experience in building NLP tools for the extraction of PDDI information [10,14] illustrated some of the difficulty and led us to the conclusion that some amount of manual involvement in the process was required.

Our goal in this study was to address two key questions in the development of human-assisted processes for curating PDDI information. Specifically, who should conduct the annotation and what sort of assistance should they receive? Although pharmacists and other domain experts familiar with drug information presumably have the background and training necessary to interpret SPLs, annotation by experts is often prohibitively difficult. Thus, we set out to gain some preliminary insight into the practicality of asking participants not specifically trained in drug information to annotate this data. Second, we were interested in understanding what level of assistance might be helpful for users. If our NLP tools were found to speed completion of annotation tasks without reducing accuracy, this would decrease the cost of PDDI annotation even for nonexperts.

### Is It Possible for Nonexperts to Produce Reliable PDDI Annotations From Drug Labels?

Annotation results from the nonexpert group were relatively strong in every scenario and better than the performance of the NLP pipeline (Figure 4). These findings suggest that nonexperts might be able to produce reliable PDDI annotations from drug labels with accuracy levels similar to those of experts and that crowdsourcing might be a feasible option for annotating PDDIs across a broader range of drug product labels. Our results are consistent with earlier demonstrations of the feasibility of applying crowdsourcing to related problems in annotation of biomedical texts [19-22].

Ensuring the success of nonexpert annotations of PDDI mentions will likely require greater attention to two keys issues: the usability of the annotation tools and the selection of the annotators.

Although self-reported task completion times (Table 2) indicated that two of the nonexperts were able to complete all tasks in times comparable to those of the expert, one nonexpert (Nonexpert 3) needed substantially more time. Differences in F_1_ scores (Table 1) suggest that annotations provide by Nonexpert 3 were of slightly lower quality than those of the other two nonexperts. The combination of increased task-completion time and lower F_1_ scores suggest that Nonexpert 3 may have struggled more than the other participants with the annotation task. In addition, difficulties with the annotation interface prevented one expert user from completing the annotation tasks.

Despite these difficulties, responses to the usability questions (Table 3) were generally positive, suggesting that usability concerns should not be insurmountable. The small sample size and self-reported time results limit our ability to develop a nuanced understanding of specific issues that might have led to increased task completion times or dissatisfaction with the user interface. Observational user studies, including think-aloud feedback from participants, would likely provide insight into usability problems, potential opportunities for redesign [39], and any difficulties associated with the longer task completion times and lower performance of Nonexpert 3.

Results from the repeated no assistance scenario (Scenario 4) do not appear to show any learning effect based on the previous three scenarios. Exposure to the preannotations in scenarios 2 and 3 may have confused participants, pointing out complexities in interpretation of the labels that might have negatively impacted performance.

Identification of individuals who are likely to produce high-quality results will be a key challenge for successful nonexpert annotation results. Although our nonexpert participants all had relevant educational backgrounds and computer experience, variations in the task completion times and F_1_ scores suggest that some participants might find PDDI annotations more approachable than others. Future nonexpert PDDI annotation recruitment might draw on experience from prior efforts in crowdsourcing which have found that appropriate screening and training of participants can help improve outcomes [40,41].

### What Is the Influence of NLP Assistance on Annotation Quality?

Most participants perceived PDDI annotation to be easier when NER preannotation was provided. However, the full NLP assistance (Scenario 3: drug mention plus PDDI preannotations) was associated with lower levels of perceived usability for both expert and nonexpert participants (Table 2). Complaints about deleting false positives were commonly expressed in the questionnaire data. These results suggest that the performance of the NLP algorithm and the presentation of NLP preannotated PDDIs might have adversely impacted participant performance. Participants suggested several possible improvements, including preannotating with NER and then presenting NLP preannotations only after a section is annotated. The purpose then would be to highlight possibly missed interactions. We think this approach would depend on an NLP algorithm with much better sentence-level performance than the algorithm used in this study.

Although exploratory, the comparison of the agreement between users, NLP preannotations, and the reference standard (tables 4 and 5) suggests several questions for future study. The expert was slightly less likely than the nonexperts to correct NLP false negatives (Table 4, column 1), possibly because the expert might have inappropriately used knowledge of the domain or applied an overly strict interpretation of the PDDI identification guidelines. The expert user was also more likely to reject a correct NLP interpretation (Table 4, column 3) and more likely to reject an incorrect NLP assertion (Table 5, column 2) suggesting that the expert user’s thought processes were somehow different than those of the nonexperts. It is also possible that the nonexpert agreement with NLP false positives might be associated with greater trust in NLP on the part of the nonexpert participants. Of course, given the small size of this study, it is entirely possible that these participant-level observations are not statistically significant. Subsequent studies involving more participants and including investigation of user thought processes—perhaps via think-aloud protocols or retrospective interviews—would be needed to understand these phenomena.

### Limitations

The generalizability of this study is limited by the small sample size; a larger study would be needed to more accurately characterize the differences between nonexperts, experts, and the NLP annotation. Another potential limitation of our study is that we could not evaluate the characteristics of label sections that might be more difficult to read and annotate by nonexperts. The experimental design attempted to address this concern by balancing the number of sections across each scenario to minimize the effect of differences in difficulty level. The study results might have been influenced by the accuracy of the NLP algorithm and the reliability and usability of the annotation user interface. Interface revisions based on usability might lead to improved performance for experts and nonexperts. Finally, as we did not conduct any debriefing interviews or otherwise assess participant mental states, we are only able to speculate as to factors that might contribute to differences in task performance.

### Conclusions

Our goal was to explore of use of nonexperts to annotate PDDI mentions in drug product labels. Our results suggest that nonexperts could produce reliable PDDI annotations from drug labels with efficiency comparable to that of an expert annotator with training in pharmacy or pharmaceutics, indicating that the task of extracting PDDIs from drug product labeling might be suitable for crowdsourcing. Although NER preannotation was found useful to both experts and nonexperts, NLP preannotation as implemented in this study seemed to present an obstacle to all participants. A high performance NLP algorithm might still be helpful if NLP preannotations are shown to annotators after a section is annotated, if only to highlight possibly missed interactions. Improvements in the usability of the annotation tool and screening of potential annotators might further increase performance.

## Appendix

Multimedia Appendix 1 Annotation guidelines provided to study participants.

Multimedia Appendix 2 Full precision, recall and F1 results for all participants and for the NLP pipeline in each of the four scenarios.

Multimedia Appendix 3 Subjective questionnaires.

Multimedia Appendix 4 Subjective responses, Scenario 1.

Multimedia Appendix 5 Subjective responses, Scenario 2.

Multimedia Appendix 6 Subjective responses, Scenario 3.

Multimedia Appendix 7 Subjective responses, Scenario 4.

## Abbreviations

NER: named entity recognizer
NLP: natural language processing
SPL: structured product label
PDDI: potential drug-drug interactions
