# Supplementary material for: Using Nonexperts for Annotating Pharmacokinetic Drug-Drug Interaction Mentions in Product Labeling: A Feasibility Study
Source: JMIR Res Protoc. 2016 Apr 11;5(2):e40. doi: 10.2196/resprot.5028 (PMC4844909; doi:10.2196/resprot.5028)
Supplement: Multimedia Appendix 1 [file resprot_v5i2e40_app1.pdf]

Annotation Guidelines

Richard D. Boyce
Yifan Ning
Andres M. Hernandez

Introduction

The purpose of this document is to provide instructions for the manual annotation of drug package inserts with information regarding drug-drug interactions. The annotation task involves marking up specific entities in the text of drug package inserts, such as mentions of drug product names and active ingredients, as well as marking up interactions between these entities. To make annotation faster, some of the entities and DDIs have already been marked up in the text using the NER (Name Entity Recognition) component and the NLP pipeline. The tool used for annotation documents is Domeo. This tool is used along with a plugin developed for annotation of pharmacokinetic drug-drug interactions. For instructions in how to use this annotation tool please see below.

Drug interactions Statements

The data used in this study consisted of 208 drug labels sections obtained from FDA-approved drugs labels in the DailyMed database used in previous projects. This dataset has a representative sample of sections that have unambiguously interacting, non-interacting drug pairs, and also no mention of interactions. To account the regulation changes about the description statements for drug labels, our corpus contents both drug labels sections prior and after year 2000.

How to Access to Domeo Tool

In Firefox or Google Chrome from a desktop or laptop computer go to:

URL: http://dbmi-icode-01.dbmi.pitt.edu:2020/Domeo/

NOTE: The tool has not been tested on tablet or phone platforms and might not work properly.

The Domeo annotation tool is an extensible web application built using Google web toolkit (GWT) that enables the creation of annotations using the Open Annotation Ontology (AO). Domeo is also able to select and execute external text mining services, as well to encode the results in the AO format.

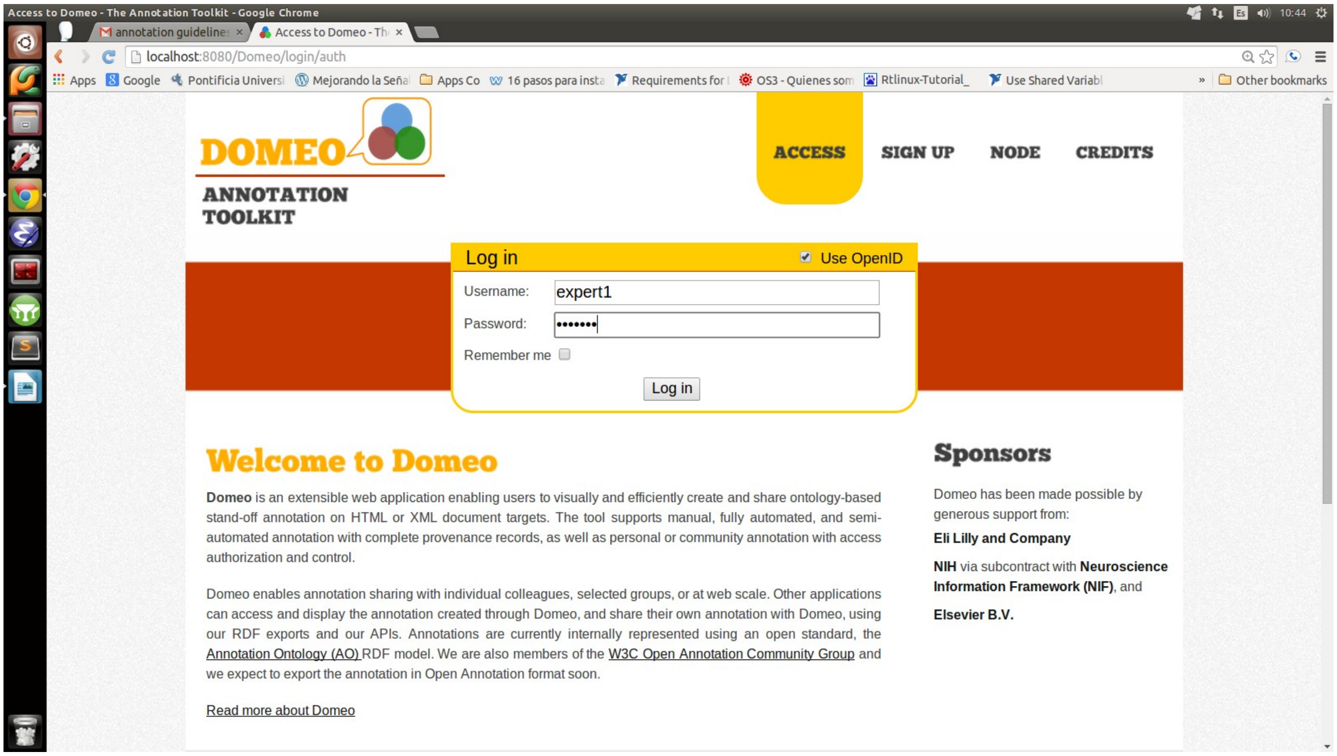

Figure 1. Domeo authentication interface

Domeo has authentication via user and password, after typing the *URL* you will see the authentication interface and after typing user and password the Domeo dashboard is showed. For accessing to the annotator interface, the tag Annotator should be clicked then the annotation main interface appears.

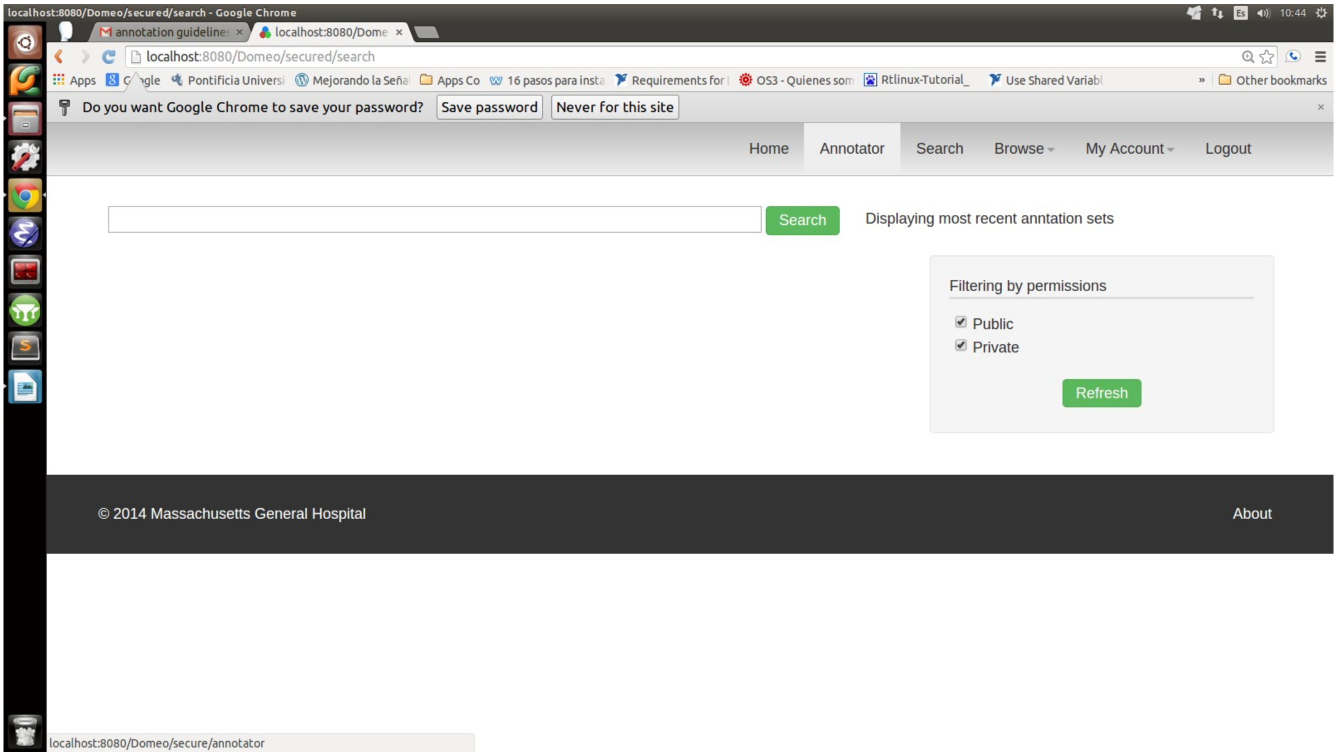

Figure 2. Domeo Dashboard

Annotating Entities

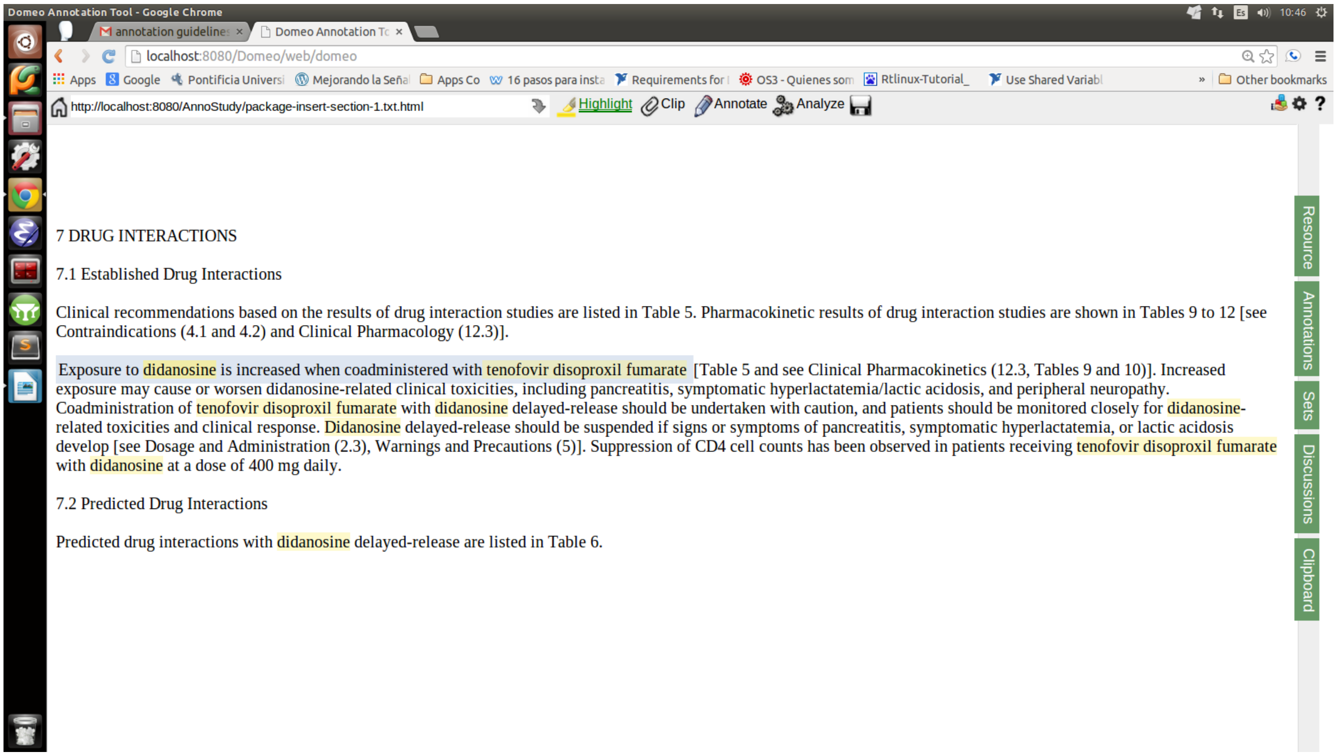

Figure 3. Domeo annotation interface

The user should type the URL in the top bar next to highlight option to access to the label that will be annotated. Then if there are previous annotations the importing option interface appears, the user should **“Select My Sets”** and then **“Import”** to retrieve the annotations. *NOTE: Not all sections will have pre-annotated entities.*

Figure 4. Domeo importing interface

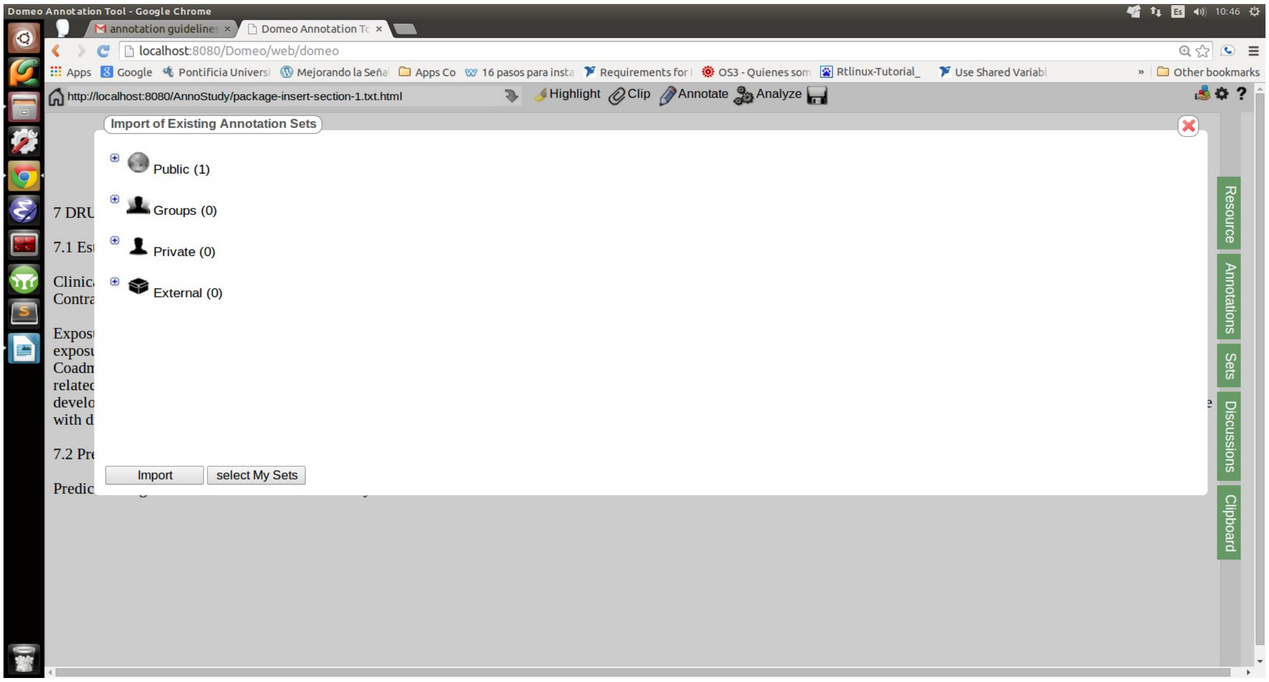

Entities are annotated by selecting a span of text along with the **Highlight** option. The first task in the annotation of DDIs is to highlight all the drug mentions in the drug product insert. There are three types of entity that need to be annotated as part of this annotation task: active ingredients, metabolites, and drug products. Each of these entities is described in more detail below, however the selection of the type of entity has to be made in the second part of the annotation task.

NOTE: highlight only these three types. Drug classes (e.g., “statins”, “anticoagulants”) or various groupings (e.g., “CYP3A inhibitors”) are out of scope for this study so please just ignore these mentions.

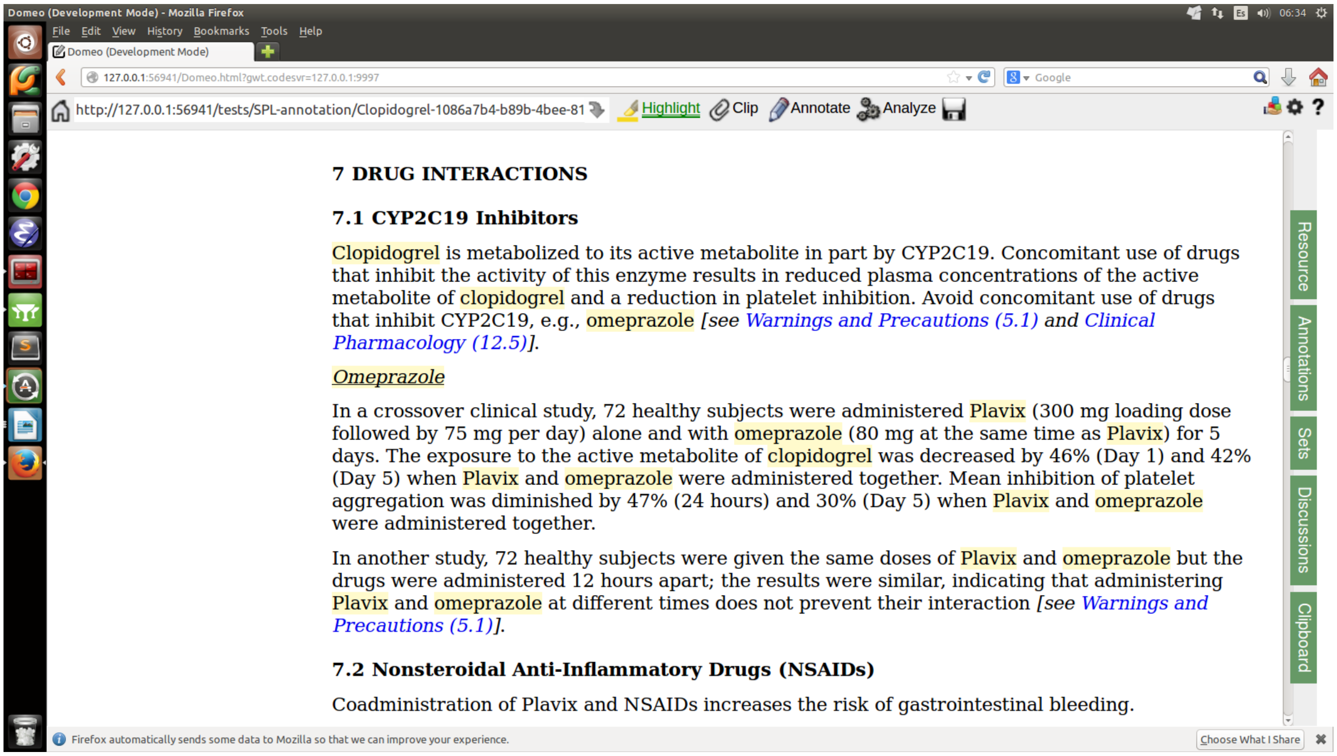

Figure 5. Highlighting entities in Domeo

The figure shows how to select entities using Domeo and the text label, first the user will select the drug label section, then Domeo will show the pre-annotations that are loaded by the NLP pipeline. Using the option **"Select My Sets"** all entities pre-annotations and DDIs will be tagged in the text. Finally using the **Highlight** tool, the user will select entities mention that were missed by the NER.

Active ingredient

An active ingredient is defined as a biologically active substance that is either synthetically manufactured or an endogenous substance extracted and processed to be reintroduced into an organism for the prevention, diagnosis, and/or treatment of disease states. There are both chemical and generic names for active ingredients. Most statements represent an active ingredient by its generic name, but they do occasionally refer to them by more precise chemical names. In either case, please use the same "Active ingredient" entity.

Mentions of active ingredients that act as modifiers in larger phrases, e.g., "cyclosporine therapy", "levonorgestrel AUC", should also be annotated – again, only annotate the text spans corresponding to just the active ingredients.

Example annotations (annotated active ingredients in bold):

- Co-administration of **tacrolimus** with **nelfinavir** increased blood concentrations of **tacrolimus** significantly.
- An open-label, randomized, three-way crossover study in 18 healthy subjects assessed the effect

of a single 800 mg oral dose of **fluconazole** on the pharmacokinetics of a single 1200 mg oral dose of **azithromycin** as well as the effects of **azithromycin** on the pharmacokinetics of **fluconazole**.

- **Cyclosporine** AUC and Cmax were determined before and after the administration of **fluconazole** 200 mg daily for 14 days in eight renal transplant patients who had been on **cyclosporine** therapy for at least 6 months and on a stable **cyclosporine** dose for at least 6 weeks.

- There was no significant difference in ethinyl estradiol or **levonorgestrel** AUC after the administration of 50 mg of DIFLUCAN.

- Co-administration of **fosamprenavir**/**ritonavir** with **paroxetine** significantly decreased plasma levels of **paroxetine** [***Note:*** in this case there are *four* active ingredient annotations, fosamprenavir, ritonavir, and paroxetine twice]

Please annotate all mentions of active ingredients in the package insert excerpts, including active ingredients that are not part of interactions. Most of the active ingredients have been automatically pre-annotated; however *the automatic process may have missed some mentions*. Please add Active ingredient annotations for these missed mentions.

Metabolite

For this analysis, a metabolite is the chemical product of some enzymatic process on an active ingredient.

Example annotations (annotated metabolites in bold, corresponding active ingredients in italics):

- The pharmacokinetics of *bupropion* and its **hydroxy metabolite** were unaffected.

- Concentrations of *thioridazine* and its two active metabolites, **mesoridazine** and **sulforidazine**, increased three-fold following co-administration of fluvoxamine.

If the active ingredient is part of the name of the metabolite, as in, for example, *erythrohydro-bupropion*, create an Active ingredient annotation for the active ingredient part of the name (*bupropion*), and select that annotation as the value for the Metabolite annotation that covers the whole name (*erythrohydro-bupropion*). ***Please annotate all mentions of metabolites in the package insert samples, including metabolites that are not participants in interactions.***

Drug product

A drug product is a manufactured compound containing one or more active ingredients and marketed under some trade name. For example, “Wellbutrin” is a drug product that contains a single active

ingredient with the generic name "bupropion."

Analogous to the annotation of active ingredients, only annotate the portion of text that corresponds to the drug product itself – do not include surrounding words and phrases that are not part of the name of the drug product.

Example annotations (annotated drug product in bold):

- Available data suggest that there is no significant effect of valproate on the clearance of **Felbatol** at steady-state.
- The mean percentage increase in the **Glucotrol** AUC after fluconazole administration was 56.9% (range: 35 to 81%).
- Coadministration of phenytoin with a dose bioequivalent to 34mg **SULAR** tablets in epileptic patients lowered the nisoldipine plasma concentrations to undetectable levels.

Please annotate all mentions of drug products in the package insert excerpts, including drug products that are not involved in interactions. Most drug products in the text have been pre-annotated automatically, but be aware that the automatic process may have missed some mentions. Please annotate mentions of drug products that have been missed.

Annotating Interactions

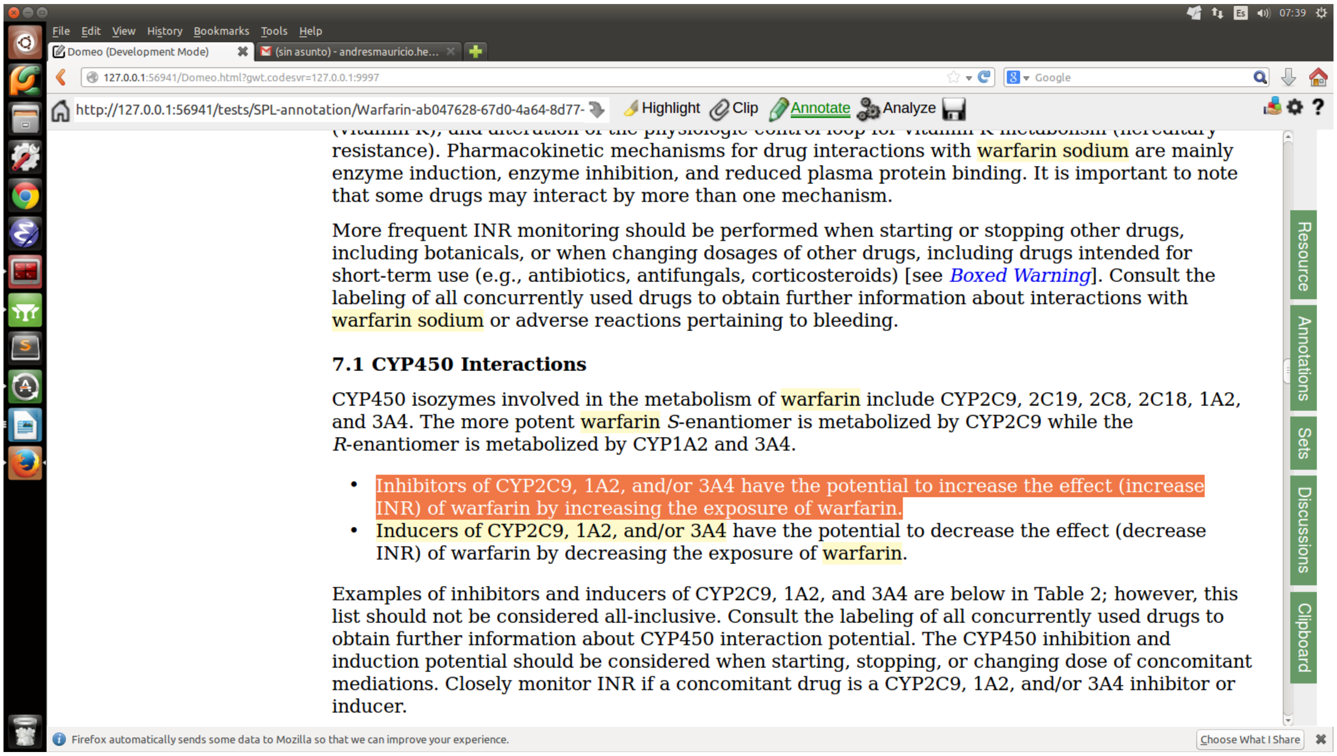

Figure 6. Annotating DDIs in Domeo

For this analysis we define a *drug-drug interaction* as any alteration of the disposition and/or effect of

an active ingredient or metabolite, owing to the presence of another active ingredient or metabolite.

The components of an interaction include a *precipitant* and an *object*:

- *precipitant* – the active ingredient or metabolite that effects an interaction.
- *object* – the active ingredient or metabolite that is altered by the interaction.

Several pieces of information are recorded as part of the annotation of a drug-drug interaction:

- The participants in the interaction: precipitant and object.
- The modality of the interaction: whether the occurrence of the interaction is affirmed (e.g., *fluconazole reduced the clearance of IV midazolam by 51%*) or negated (e.g., *there was no significant pharmacokinetic interaction between fluconazole and azithromycin*).
- The "textual evidence" for the interaction, referred to as "interaction phrase": words or phrases that are used in the text to describe interactions, e.g., *reduced the clearance of IV midazolam by 51%, the AUC and Cmax of glyburide (5 mg single dose) were significantly increased*.
- The type of the interaction statement: quantitative statements provide numerical data about the effects or clearly state "no effect" while qualitative statements provide no numerical data.

The next section defines more specifically the types of statements that qualify as drug-drug interaction statements. Then follows instruction on how to annotate such statements using Domeo.

Definition of an Interaction Statement

A *drug interaction statement* is defined as a sentence or phrase published in an FDA drug package insert that describes an affirmed or negated **pharmacokinetic** *drug-drug interaction* which reports the effect on absorption, distribution, metabolism, or elimination of one drug or its metabolite(s) on another drug or metabolite(s). An affirmed interaction states that an interaction occurs between two drugs while a negated interaction states that there is no interaction between two drugs.

NOTE: Since pharmacokinetics focuses on absorption, distribution, metabolism, or elimination of a drug, the following terms are quite common in these statements (not an exhaustive list):

Cl (clearance), AUC (Area Under the Concentration Time curve), F (fraction absorbed), T1/2 (half-life), Cmax (maximum concentration), inhibits, induces.

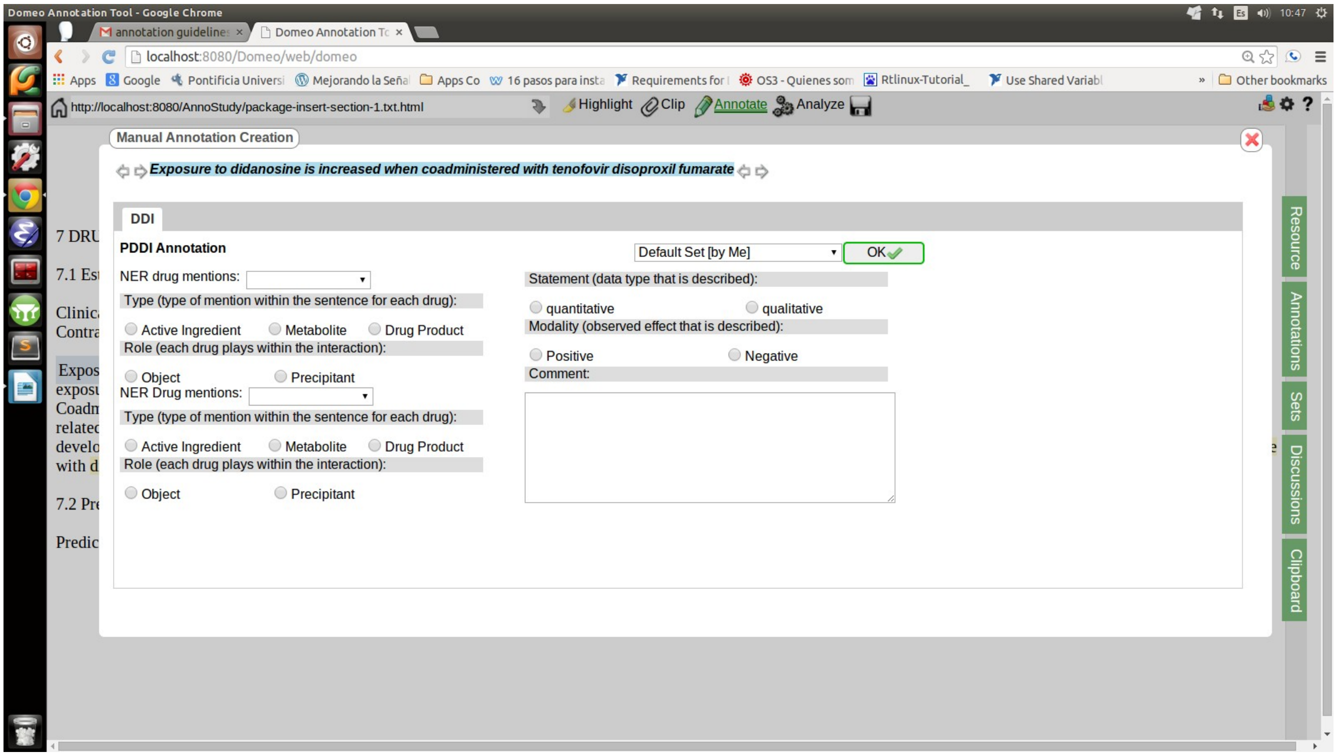

Figure 7. DDI Form

As you are annotating package insert sections, you will come across two types of statements:

- *Interaction statements* - defined as statements that describe an affirmed or negated *pharmacokinetic* interaction between two drugs.
- *Non-interaction statements* - defined as statements that mention two different drugs but do not describe an affirmed or negated *pharmacokinetic* interaction between the two drugs.

Notice that both types of statements include mention of two or more drugs, but only the interaction statement describes an affirmed or negated pharmacokinetic drug-drug interaction.

Interaction statements are divided into two subtypes: *quantitative* and *qualitative* statements. *Quantitative interaction statements* contain quantitative data regarding the affirmed or negated interaction, dosing information, or other clinical study parameters such as study length and number of participants in the study. *Qualitative interaction statements* come in two styles. The first is a statement in which an affirmed or negated pharmacokinetic drug-drug interaction is reported but *no quantitative data* is given. The second kind of qualitative statement is a

statement that *predicts* an affirmed or negated pharmacokinetic drug-drug interaction without giving any data. There are no subtypes of *non-interaction statements*.

Thus, the three types of interaction statements are *Quantitative interaction*, *Qualitative interaction*, and *Non-interaction* statements. Examples of each type of interaction statement are below:

Interaction examples

(<u>Quantitative</u>)

In the following examples, quantitative data is given regarding a change in both the Cmax and AUC of the object drug while also including dosing information.

- Similarly, following administration of 1 gram of erythromycin ethyl succinate and 200 mg itraconazole as single doses, the mean Cmax and AUC0-∞ of itraconazole increased by 44% (90% CI: 119% to 175%) and 36% (90% CI: 108% to 171%), respectively.
- In a study in healthy volunteers, coadministration of buspirone (30 mg as a single dose) with rifampin (600 mg/day for 5 days) decreased the plasma concentrations (83.7% decrease in Cmax; 89.6% decrease in AUC) and pharmacodynamic effects of buspirone.

In the next set of examples, quantitative data is given with little or no reference to study parameters.

- Coadministration of buspirone with cimetidine was found to increase Cmax (40%) and Tmax (2-fold), but had minimal effects on the AUC of buspirone.
- Grapefruit juice given to healthy volunteers increased amiodarone AUC by 50% and Cmax by 84%, and decreased DEA to unquantifiable concentrations.
- Amiodarone taken concomitantly with digoxin increases the serum digoxin concentration by 70% after one day.
- Since the concomitant administration of warfarin with amiodarone increases the prothrombin time by 100% after 3 to 4 days, the dose of the anticoagulant should be reduced by one-third to one-half, and prothrombin times should be monitored closely.

The next set of examples contain quantitative data on both the effect of a drug on another drug and the clinical trial from which the data comes; including dosing information, study participants, and study length.

- When zidovudine (100 mg q3h ×5) was coadministered with daily azithromycin (600 mg, n=5 or 1200 mg, n=7), mean Cmax, AUC and Clr increased by 26% (CV 54%), 10% (CV 26%) and 38% (CV 114%), respectively.

- In a placebo-controlled study, saquinavir administered as a 1200 mg dose, tid, for 5 days (n=12), a 56% reduction in the clearance of midazolam following a single 0.05 mg/kg IV dose was observed.
- In a study of 11 women with bipolar disorder receiving lithium carbonate at a dosage of 600 mg to 1200 mg/day, administration of 100 mg flurbiprofen every 12 hours increased plasma lithium concentrations by 19%.
- Following co-administration of tacrolimus and sirolimus (2 or 5 mg/day) in stable renal transplant patients, mean tacrolimus AUC0-12 and Cmin decreased approximately by 30% relative to tacrolimus alone.

The following (final) example is more complex. While this interaction statement reports that there is no effect on pharmacokinetics, this statement is still considered to be quantitative because it describes a negated interaction and includes clinical study information (dosing, length, subjects).

- Administration of a 600 mg single oral dose of azithromycin had no effect on the pharmacokinetics of efavirenz given at 400 mg doses for 7 days to healthy adult subjects.

(<u>Qualitative</u>)

The following are examples of the first type of qualitative interaction statements. Each statement reports on an interaction but does not give any quantitative data.

- Fluconazole increases the serum concentrations of theophylline.
- Cyclosporine significantly increased rosuvastatin exposure.
- The combination of lopinavir and ritonavir significantly increased rosuvastatin exposure.
- Concomitant administration of itraconazole and cyclosporine or tacrolimus has led to increased plasma concentrations of these immunosuppressants.
- Aluminum- and magnesium-containing antacids reduce the peak serum levels (rate) but not the AUC (extent) of azithromycin (500 mg) absorption.

The next set of examples display the second type of qualitative interaction statements. Each of the statements predict that an interaction may occur, but do not give any quantitative data to support.

- Erythromycin and clarithromycin (and possibly other macrolide antibiotics) and tetracycline may increase digoxin absorption.
- Calcium, particularly if administered rapidly by the intravenous route, may produce serious arrhythmias in digitalized patients.
- Rifampin may decrease serum digoxin concentration, especially in patients with renal dysfunction, by increasing the non-renal clearance of digoxin.
- Inducers of CYP3A4 may decrease the plasma concentrations of itraconazole.

- Potentiation of warfarin-type (CYP2C9 and CYP3A4 substrate) anticoagulant response is almost always seen in patients receiving amiodarone and can result in serious or fatal bleeding.
- Inhibitors of CYP3A4 such as erythromycin, clarithromycin, ketoconazole, itraconazole, ritonavir and grapefruit juice may increase plasma concentrations of estrogens and may result in side effects.

These last examples show statements that imply interactions, but do not give quantitative data.

- Cisapride, oral midazolam, nisoldipine, pimozide, quinidine, dofetilide, triazolam and levacetylmethadol (levomethadyl) are contraindicated with itraconazole.
- Ergot alkaloids metabolized by CYP3A4 such as dihydroergotamine, ergometrine (ergonovine), ergotamine and methylergometrine (methylergonovine) are contraindicated with itraconazole.
- Caution is advised when midazolam is administered concomitantly with drugs that are known to inhibit the P450-3A4 enzyme system such as cimetidine (not ranitidine), erythromycin, diltiazem, verapamil, ketoconazole and itraconazole.
- Coadministration of cisapride is contraindicated in patients receiving fluconazole.

Non-interaction examples

The first set of examples for non-interaction statements all include two or more drugs, but do not describe an affirmed or negated pharmacokinetic drug-interaction.

- An increased risk of congenital malformations is associated with the use of diazepam and chlordiazepoxide during the first trimester of pregnancy has been suggested in several studies.
- Erythromycin and clarithromycin are substrates and inhibitors of the 3A isoform subfamily of the cytochrome P450 enzyme system (CYP3A).
- The use of live vaccines should be avoided; live vaccines may include, but are not limited to measles, mumps, rubella, oral polio, BCG, yellow fever, and TY 21a typhoid.
- These include, but are not limited to, aminoglycosides, amphotericin B, and cisplatin.
- Midazolam HCl syrup is a benzodiazepine and is a Schedule IV controlled substance that can produce drug dependence of the diazepam-type.
- CRESTOR was compared with the HMG-CoA reductase inhibitors atorvastatin, simvastatin, and pravastatin in a multicenter, open-label, dose-ranging study of 2,240 patients with hyperlipidemia or mixed dyslipidemia.
- Although clinical studies have shown that CRESTOR alone does not reduce basal plasma cortisol concentration or impair adrenal reserve, caution should be exercised if CRESTOR is administered concomitantly with drugs that may decrease the levels or activity of endogenous steroid hormones such as ketoconazole, spironolactone, and cimetidine.

- Carbamazepine, phenobarbital, and phenytoin are all inducers of CYP3A4.
- Edema has been reported in patients concomitantly receiving SPORANOX® and dihydropyridine calcium channel blockers.
- Controlled studies in animals and man have shown no stimulation of any pituitary hormone by ranitidine and no antiandrogenic activity, and cimetidine-induced gynecomastia and impotence in hypersecretory patients have resolved when ranitidine has been substituted.
- It appears possible, although there is no supporting experimental evidence, that the high renal clearance of desglymidodrine (a base) is due to active tubular secretion by the base-secreting system also responsible for the secretion of such drugs as metformin, cimetidine, ranitidine, procainamide, triamterene, flecainide and quinidine.

These next examples display statements that include quantitative data, but do not describe an affirmed or negated interaction, and hence are non-interaction statements.

- Two randomized, controlled studies for the treatment of oropharyngeal candidiasis have been conducted (total n=344). In one trial, clinical response to either 7 or 14 days of itraconazole oral solution, 200 mg/day, was similar to fluconazole tablets and averaged 84% across all arms.
- In Study 174, more subjects discontinued from the combination of azithromycin and rifabutin (22.7%) than from azithromycin alone (13.5%; p=0.026) or rifabutin alone (15.9%; p=0.209).

How to annotate interaction phrases

To annotate an interaction, create an annotation from the annotation option in Domeo. Next, fill the form of the DDI plugin that is showed by the annotation tool. Each Interaction annotation has eight fields, all of which require a value: Drug1, Drug1 Role (Precipitant or Object), Drug 1 type (Active Ingredient, Metabolite or Drug Product), Drug 2, Drug 2 Role, Drug 2 Type, Statement Type, and Modality. These slots are discussed below.

The Precipitant and Object slots of an **Interaction** annotation refer to the participants of the drug-drug interaction that is annotated. The values of precipitant and object exist Active ingredient, Metabolite, or Drug product. Both the Precipitant slot and the Object slot can have one and only one annotation as their value.

- The effect of *fluconazole*$_1$ on the pharmacokinetics and pharmacodynamics of *midazolam*$_1$ was examined in a randomized, cross-over study in 12 volunteers. In the study, subjects ingested placebo or 400 mg *fluconazole*$_2$ on Day 1 followed by 200 mg daily from Day 2 to Day 6. In addition, a 7.5 mg dose of *midazolam*$_2$ was orally ingested on the first day, 0.05 mg/kg was administered intravenously on the fourth day, and 7.5 mg orally on the sixth day. *Fluconazole*$_3$ reduced the clearance of IV *midazolam*$_3$ by 51%.

Example annotations:

- Concomitant administration of cimetidine and venlafaxine in a steady-state study for both drugs resulted in inhibition of first-pass metabolism of venlafaxine in 18 healthy subjects.

- Coadministration of tacrine with theophylline increased theophylline elimination half-life and average plasma theophylline concentrations by approximately 2-fold.

- In a study of 13 healthy, male volunteers, a single 40 mg dose of tacrine added to fluvoxamine 100 mg/day administered at steady-state was associated with five- and eight-fold increases in tacrine Cmax and AUC, respectively, compared to the administration of tacrine alone.

- In healthy volunteers, co-administration with gemfibrozil (600 mg twice daily for 3 days) resulted in an 8.1-fold (range 5.5- to 15.0- fold) higher repaglinide AUC and a 28.6-fold (range 18.5- to 80.1-fold) higher repaglinide plasma concentration 7 hours after the dose.

- The AUC and Cmax of glyburide (5 mg single dose) were significantly increased following the administration of fluconazole in 20 normal male volunteers.

- Following oral administration of two 150-mg Bupropion Hydrochloride Extended-Release Tablets, USP (SR) with and without 800 mg of cimetidine, the pharmacokinetics of bupropion and its hydroxy metabolite were unaffected.

- There was no significant pharmacokinetic interaction between fluconazole and azithromycin.

The **Modality** slot of an Interaction annotation is used to capture whether an interaction described in the text is affirmed or negated. A sentence stating that an interaction does in fact occur, e.g., *the AUC and Cmax of glyburide (5 mg single dose) were significantly increased following the administration of fluconazole in 20 normal male volunteers*, corresponds to an affirmed interaction; a sentence explicitly stating that an interaction does not occur, e.g., *co-administration of metoclopramide with the long-acting propranolol did not have a significant effect on propranolol's pharmacokinetics*, corresponds to a negated interaction.

The two possible values for the **Modality** slot are **Positive**, for affirmed interactions, and **Negative**, for negated interactions.

Please annotate all quantitative or quantitative interaction phrases in the package insert samples. Interactions that are not explicitly asserted (or negated) but whose presence (or absence) can be inferred from a sentence should not be annotated. For example, the sentence, *fluvoxamine and thioridazine should not be co-administered*, does not count as a drug-drug interaction for the purposes of this annotation task.

Also, mere descriptions of studies do not justify the annotation of an interaction phrase. For example, the sentence below does not contain any information about the presence or absence of an interaction between the active ingredients mentioned; thus, no interaction phrase should be created for this sentence.

- An open-label, randomized, three-way crossover study in 18 healthy subjects assessed the effect of a single 800 mg oral dose of **fluconazole** on the pharmacokinetics of a single 1200 mg oral

dose of **azithromycin** as well as the effects of **azithromycin** on the pharmacokinetics of **fluconazole**.

Elaborations of observed effects that do not mention the precipitant and object of an interaction should be annotated as an interaction phrase so long *if they provide quantitative data*. Thus, in the following statement, the entire interaction phrase needs to be annotated.

- The AUC and Cmax of glyburide (5 mg single dose) were significantly increased following the administration of fluconazole in 20 normal male volunteers. There was a mean ± SD increase in AUC of 44% ± 29% (range: 13 to 115%) and Cmax increased 19% ± 19% (range: 23 to 62%).

Example annotations (precipitants in italic with subscript *p*, objects in italic with subscript *o*, interaction phrases in bold, modality indicated following sentence):

- The AUC and Cmax of *glyburide*$_o$ (5 mg single dose) were significantly increased following the administration of *fluconazole*$_p$ in 20 normal male volunteers. [Modality: Positive]

- There was no significant pharmacokinetic interaction between *fluconazole*$_p$ and *azithromycin*$_0$. [Modality: Negative]

- Coadministration of *tacrine*$_p$ with *theophylline*$_o$ increased theophylline elimination half-life and average plasma theophylline concentrations by approximately 2-fold. [Modality: positive]

- In a study of 13 healthy, male volunteers, a single 40 mg dose of *tacrine*$_o$ added to *fluvoxamine*$_p$ 100 mg/day administered at steady-state was associated with five- and eight-fold increases in tacrine Cmax and AUC, respectively, compared to the administration of tacrine alone. [Modality: Positive]

- In healthy volunteers, co-administration with *gemfibrozil*$_p$ (600 mg twice daily for 3 days) resulted in an 8.1-fold (range 5.5- to 15.0- fold) higher repaglinide AUC and a 28.6-fold (range 18.5- to 80.1-fold) higher *repaglinide*$_o$ plasma concentration 7 hours after the dose. [Modality: Positive]

Note that the sentence from the last example contains two mentions of *repaglinide*. ***As a heuristic, pick the last one as the object of the interaction in situations like these.***

An example of a sentence containing two interactions:

- In this placebo-controlled, double-blind, randomized, two-way crossover study carried out over three cycles of oral contraceptive treatment, *fluconazole*$_p$ dosing resulted in small increases in the mean AUCs of *ethinyl estradiol*$_o$ and *norethindrone*$_o$ compared to similar placebo dosing.

The two Interaction annotations for the sentence above are:

1. Precipitant: *fluconazole*; Object: *ethinyl estradiol*; Modality: Positive; Interaction phrase: resulted in small increases in the mean AUCs

2. Precipitant: *fluconazole*; Object: *norethindrone*; Modality: Positive; Interaction phrase: resulted in small increases in the mean AUCs

A more complex example:

- Concomitant administration of *cimetidine*$_p$ and *venlafaxine*$_{o,1}$ in a steady-state study for both drugs resulted in inhibition of first-pass metabolism of venlafaxine in 18 healthy subjects. The oral clearance of *venlafaxine*$_{o,2}$ was reduced by about 43%, and the exposure (AUC) and maximum concentration (Cmax) of the drug were increased by about 60%.

The statement above describes the same interaction twice. Both mentions should be annotated:

1. Precipitant: *cimetidine*$_p$; Object: *venlafaxine*$_{o,1}$; Modality: Positive; Interaction phrase: resulted in inhibition of first-pass metabolism

2. Precipitant: *cimetidine*$_p$; Object: *venlafaxine*$_{o,2}$; Modality: Positive; Interaction phrase: The oral clearance of *venlafaxine*$_{o,2}$ was reduced by about 43%, and the exposure (AUC) and maximum concentration (Cmax) of the drug were increased by about 60%

Another complex example:

- Following oral administration of two 150-mg Bupropion Hydrochloride Extended-Release Tablets, USP (SR) with and without 800 mg of *cimetidine*, the pharmacokinetics of *bupropion* and its *hydroxy metabolite* were unaffected. However, there were 16% and 32% increases, respectively, in the AUC and Cmax of the combined moieties of *threohydro*- and *erythrohydro-bupropion*.
