# Supplementary material for: Using Nonexperts for Annotating Pharmacokinetic Drug-Drug Interaction Mentions in Product Labeling: A Feasibility Study
Source: JMIR Res Protoc. 2016 Apr 11;5(2):e40. doi: 10.2196/resprot.5028 (PMC4844909; doi:10.2196/resprot.5028)
Supplement: Multimedia Appendix 2 [file resprot_v5i2e40_app2.pdf]

Multimedia Appendix 2

Annotator	Metric	Scenario 1	Scenario 2	Scenario 3	Scenario 4	Overall
**Expert**	precision	0.79	0.78	0.68	0.71	0.73
	recall	0.82	0.81	0.45	0.61	0.63
	F_1	0.80	0.79	0.54	0.66	0.68
**Non-expert 1**	precision	0.77	0.86	0.66	0.66	0.73
	recall	0.80	0.80	0.53	0.45	0.60
	F_1	0.79	0.83	0.59	0.53	0.66
**Non-expert 2**	precision	0.73	0.59	0.54	0.73	0.64
	recall	0.80	0.81	0.61	0.68	0.71
	F_1	0.76	0.68	0.57	0.70	0.67
**Non-expert 3**	precision	0.70	0.54	0.53	0.62	0.59
	recall	0.79	0.72	0.54	0.63	0.65
	F_1	0.74	0.62	0.53	0.62	0.61
**NLP**	precision	0.45	0.42	0.40	0.38	0.40
	recall	0.80	0.39	0.42	0.58	0.53
	F_1	0.58	0.40	0.41	0.46	0.46

Supplemental Table 1: Full Precision, Recall, and F1 measures for all participants and NLP system across all scenarios and overall. Scenario 1 – No Assistance. Scenario 2 – Pre-annotation of drug mentions. Scenario 3- Pre annotation of drug mentions and PDDIs. Scenario 4 – No assistance.
