# Supplementary material for: Using Nonexperts for Annotating Pharmacokinetic Drug-Drug Interaction Mentions in Product Labeling: A Feasibility Study
Source: JMIR Res Protoc. 2016 Apr 11;5(2):e40. doi: 10.2196/resprot.5028 (PMC4844909; doi:10.2196/resprot.5028)
Supplement: Multimedia Appendix 3 [file resprot_v5i2e40_app3.pdf]

Multimedia Appendix 3

Scenario 1 Questionnaire

1.1 Approximately how long did it take you to get familiar with the annotation tool for doing this section of the task?

- Less than 1 hour
- Between 1 and 3 hours
- Between 3 and 5 hours
- More than 5 hours

1.2 Please indicate your level of agreement with the following statements: I learned to use Domeo quickly

- Strongly disagree
- Disagree
- Neither
- Agree
- Strongly Agree

1.3 Please indicate your level of agreement with the following statements: I feel that I need technical support in order to use Domeo

- Strongly disagree
- Disagree
- Neither
- Agree
- Strongly Agree

1.4 How comfortable do you feel in using Domeo?

- Very comfortable
- Moderately comfortable
- Slightly comfortable
- Not at all comfortable

1.5 How easy or difficult did you find Domeo to use? (*Results of this question reported in Table 2 of paper)

- Very easy
- Moderately easy
- Neither easy nor difficult
- Moderately difficult
- Very difficult

1.6 I can effectively annotate active ingredients, metabolites , and drug products using Domeo.

- Strongly disagree

- Disagree
- Neither
- Agree
- Strongly Agree

1.7 I can effectively annotate drug-drug annotations using Domeo

- Strongly disagree
- Disagree
- Neither
- Agree
- Strongly Agree

1.8 If I make an error, I can easily recover from it

- Strongly disagree
- Disagree
- Neither
- Agree
- Strongly Agree

1.9 I could easily identify the precipitant and object of drug-drug interactions

- Strongly disagree
- Disagree
- Neither
- Agree
- Strongly Agree

10. I could easily identify the modality of drug interaction statements

- Strongly disagree
- Disagree
- Neither
- Agree
- Strongly Agree

11. I could easily identify if drug interaction statements were quantitative or qualitative

- Strongly disagree
- Disagree
- Neither
- Agree
- Strongly Agree

12. In the area below, please fill in any suggestions you have for improving the Domeo annotation tool

Scenario 2 Questionnaire

2.1 Approximately how long did it take you to get familiar with the annotation tool for doing this section of the task?

- Less than 1 hour
- Between 1 and 3 hours
- Between 3 and 5 hours
- More than 5 hours

2.2 The pre-annotated active ingredients, metabolites, and drug products provided in this stage made my work go more quickly.

- Strongly disagree
- Disagree
- Neither
- Agree
- Strongly Agree

2.3 The pre-annotated active ingredients, metabolites and drug products provided in this stage were generally accurate

- Strongly disagree
- Disagree
- Neither
- Agree
- Strongly Agree

2.4 The pre-annotated active ingredients, metabolites and drug products provided in this stage were complete

- Strongly disagree
- Disagree
- Neither
- Agree
- Strongly Agree

2.5 How easy or difficult did you find Domeo to use when pre-annotated active ingredients, metabolites and drug products were provided? (*Results of this question reported in Table 2 of paper)

- Very easy
- Moderately easy
- Neither easy nor difficult
- Moderately difficult

- Very difficult

2.6 It was easy for me to find and correct pre-annotated active ingredients, metabolites and drug products

- Very easy
- Moderately easy
- Neither easy nor difficult
- Moderately difficult
- Very difficult

2.7 I could easily identify the precipitant and object of drug interaction statements in this stage

- Strongly disagree
- Disagree
- Neither
- Agree
- Strongly Agree

2.8 I could easily identify the modality of drug interaction statements in this stage

- Strongly disagree
- Disagree
- Neither
- Agree
- Strongly Agree

2.9 I could easily identify if drug interaction statements were quantitative or qualitative in this stage

- Strongly disagree
- Disagree
- Neither
- Agree
- Strongly Agree

2.10 In the area below, please fill in any suggestions you have for improving the Domeo annotation tool

Scenario 3 Questionnaire

3.1 Approximately how long did it take you to get familiar with the annotation tool for doing this section of the task?

- Less than 1 hour
- Between 1 and 3 hours
- Between 3 and 5 hours
- More than 5 hours

3.2 The pre-annotated active ingredients, metabolites, and drug products provided in this stage made my work go more quickly.

- Strongly disagree
- Disagree
- Neither
- Agree
- Strongly Agree

3.3 The pre-annotated drug interactions provided in this stage made my work go more quickly.

- Strongly disagree
- Disagree
- Neither
- Agree
- Strongly Agree

3.4 The pre-annotated active ingredients, metabolites and drug products provided in this stage were generally accurate

- Strongly disagree
- Disagree
- Neither
- Agree
- Strongly Agree

3.5 The pre-annotated active ingredients, metabolites and drug products provided in this stage were generally complete

- Strongly disagree
- Disagree
- Neither
- Agree
- Strongly Agree

3.6 The pre-annotated drug interactions provided in this stage were generally accurate with respect to sentences highlighted, drugs involved, and the modality of the interaction statement

- Strongly disagree
- Disagree
- Neither
- Agree
- Strongly Agree

3.7 The pre-annotated drug interactions provided in this stage were generally complete with respect to sentences highlighted, drugs involved, and the modality of the interaction statement

- Strongly disagree
- Disagree
- Neither
- Agree
- Strongly Agree

3.8 How easy or difficult did you find Domeo to use when both drug interactions and active ingredients, metabolites and drug products were pre-annotated? (*Results of this question reported in Table 2 of paper)

- Very easy
- Moderately easy
- Neither easy nor difficult
- Moderately difficult
- Very difficult

3.9 It was easy for me to find and correct pre-annotated active ingredients, metabolites and drug products

- Strongly disagree
- Disagree
- Neither
- Agree
- Strongly Agree

3.10 It was easy for me to find and correct pre-annotated drug interactions

- Strongly disagree
- Disagree
- Neither
- Agree
- Strongly Agree

3.11 I could easily identify the precipitant and object of drug interaction statements in this stage

- Strongly disagree

- Disagree
- Neither
- Agree
- Strongly Agree

3.12 I could easily identify the modality of drug interaction statements in this stage

- Strongly disagree
- Disagree
- Neither
- Agree
- Strongly Agree

3.13 I could easily identify if drug interaction statements were quantitative or qualitative in this stage

- Strongly disagree
- Disagree
- Neither
- Agree
- Strongly Agree

3.14 In the area below, please fill in any suggestions you have for improving the Domeo annotation tool

Scenario 4 Questionnaire

4.1 I could easily identify the precipitant and object of drug interaction statements in this stage

- Strongly disagree
- Disagree
- Neither
- Agree
- Strongly Agree

4.2 I could easily identify the modality of drug interaction statements in this stage

- Strongly disagree
- Disagree
- Neither
- Agree
- Strongly Agree

4.3 I could easily identify if drug interaction statements were quantitative or qualitative in this stage

- Strongly disagree
- Disagree
- Neither
- Agree
- Strongly Agree

4.4 I felt that the pre-annotations provided in the previous stages helped me to learn how to annotate active ingredients, metabolites, and drug products

- Strongly disagree
- Disagree
- Neither
- Agree
- Strongly Agree

4.5 I felt that the pre-annotations provided in the previous stages helped me to learn how to annotate drug interaction statements

- Strongly disagree
- Disagree
- Neither
- Agree
- Strongly Agree

4.6 In the area below, please fill in any suggestions you have for improving the Domeo annotation tool

4.7 List the most negative aspect(s):

4.8 List the most positive aspect(s):
